# Comparative metabolomics profiling of isogenic KRAS wild type and mutant NSCLC cells in vitro and in vivo

**DOI:** 10.1038/srep28398

**Published:** 2016-06-22

**Authors:** Laura Brunelli, Elisa Caiola, Mirko Marabese, Massimo Broggini, Roberta Pastorelli

**Affiliations:** 1 Protein and Gene Biomarkers Unit, Laboratory of Mass Spectrometry, Department of Environmental Health Sciences, IRCCS-Istituto di Ricerche Farmacologiche “Mario Negri”, Milan, Italy; 2 Laboratory of Molecular Pharmacology, Department of Oncology, IRCCS-Istituto di Ricerche Farmacologiche “Mario Negri”, Milan, Italy

## Abstract

Oncogenes induce metabolic reprogramming on cancer cells. Recently, G12C KRAS mutation in isogenic NSCLC cell line has been shown to be a key player in promoting metabolic rewiring mainly through the regulation of glutamine metabolism to fuel growth and proliferation. Even though cell lines possessing many of the genetic backgrounds of the primary cancer they derive from could be a valuable pre-clinical model, they do not have the additional complexity present in the whole tumor that impact metabolism. This preliminary study is aimed to explore how cancer cell metabolism in culture might recapitulate the metabolic alterations present *in vivo*. Our result highlighted that the gross metabolic changes observed in G12C KRAS mutant cells growing in culture were also maintained in the derived xenograft model, suggesting that a simple *in vitro* cell model can give important insights into the metabolic alterations induced by cancer. This is of relevance for guiding effective targeting of those metabolic traits that underlie tumor progression and anticancer treatment responses.

Cell culture studies have revealed how different oncogenic mutations and nutrients impact on cancer metabolism. Studies using *in vitro* culture systems have led to important insights regarding nutrient utilization and the regulation of metabolic pathways by describing how cancer cells exploit existing metabolic programs to fuel proliferation and survival. In particular it has been demonstrated that KRAS activation supports the decoupling of glycolysis and TCA metabolism, with glutamine supplying increased carbon to drive the TCA cycle in different cancer cell lines such as NSCLC, pancreatic and colon^1^,^2^,^3^,^4^. These results provide evidence that oncogenic KRAS is involved in the metabolic reprogramming of cancer cells. Cell lines possessing many of the genetic and genomic alterations found in primary lung cancer and/or representing diverse lung cancer subtypes, as in our case, are of values as pre-clinical models. Nevertheless cancer cell lines display potential limitations that must be taken into account for metabolic study. Indeed, the study of tumor metabolism *in vivo* invariably introduces new complexities, but has, on the other hand, the great advantage of taking into consideration how the whole tumor heterogeneity and the interaction between different cell types as well as nutrient availability impact on its metabolism^5^. Moreover, tumor cells also have metabolic interactions with normal host tissues that could have a profound effect on cancer metabolism.

So far limited data exist that address how faithfully cancer metabolism in culture recapitulate the metabolic alterations observed in cancer models *in vivo*.

In this explorative investigation we applied a quantitative metabolomics profiling to human NSCLC isogenic cell lines harboring G12C or WT KRAS isoforms and to their derived tumors implanted in immunodeficent mice, in order to decipher whether and how much the cancer cell metabolic findings obtained *in vitro* could be translated into the metabolism of the whole tumor.

## Results and Discussion

With the aim of better understanding how whole-animal physiology impacts on metabolic alteration observed in *in-vitro* culture systems, we investigated the KRAS associated metabolomic signatures on NSCLC cell lines harboring G12C mutation or WT KRAS both in monolayer culture and in corresponding tumors derived NSCLC xenograft models.

The clones had similar growth rate *in vitro*^6^ and, when subcutaneously transplanted in nude mice, displayed comparable tumor growth rate (Supplemental Figure S1). We then checked for the percentage of murine infiltrating cells in tumor xenografts (of human origin), analyzing the DNA by Real-Time PCR with species-specific probes. We found that the human component constituted about 85.1% (average of the eight samples), so murine fraction was negligible. Importantly, the two clones had similar percentages of infiltrating murine cells (13.7% for WT and 16.1% for G12C KRAS xenografts) thus allowing a proper comparison of the two systems *in vivo*.

On the basis of the results obtained in our recent publication^1^ using an untargeted mass spectrometry-based metabolomics approach, we focused our attention on G12C KRAS mutant because not only this mutation showed the most marked metabolic remodelling among the investigated KRAS mutants clones (G12D and G12V), but also because G12C is the most representative mutation in NSCLC patients. To better characterize the metabolic alterations induced by G12C KRAS mutation, we applied a mass spectrometry-based targeted quantitative approach allowing simultaneous quantification of 188 metabolites on a well-defined isogenic system derived from the human NSCLC cell line NCI-H1299, expressing the WT or the G12C mutant KRAS. The study has been conducted both *in vitro* in cells growing in monolayer (nine biological replicates) as well as on their derived tumor xenograft models (four biological replicates) from which metabolite extracts for each condition were analysed.

Concentrations of metabolites passing our filtering criteria in cell systems and xenograft model are listed as µM in Supplemental Tables S1 and S2.

**Figure 1:**
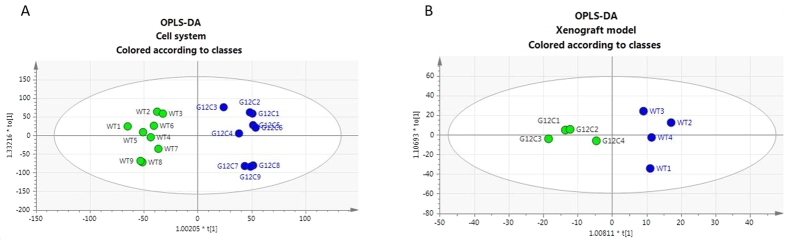
OPLS-DA analysis of the quantified metabolites in NSCLC cell lines harboring G12C and WT KRAS isoforms and their relative derived xenograft tumor model. Panel A, OPLS-DA score plot showing classes separated according to their metabolic signature, where classes correspond to NSCLC cell lines harboring G12C or WT isoforms. Panel B, OPLS-DA score plot showing classes separated according to their metabolic signature, where classes correspond to xenograft models derived from NSCLC G12C or WT isoforms cell lines.

To gain a deeper understanding of the capability to translate our cell monolayer metabolic fingerprint to the biochemistry of whole tumor, an orthogonal projection to latent structures-discriminant analysis (OPLS-DA) was used. OPLS-DA models demonstrated robust group separation between G12C and WT KRAS isoforms both in cell system (R2Ycum = 0.961; Q2Ycum = 0.997) and xenograft model (R2Ycum = 0.909, Q2Ycum = 0.994) as shown in Fig. 1A,B. Focusing on the metabolites that strongly contributed to discriminate G12C KRAS mutation from WT counterpart in monolayer cell system and in xenograft tumor (S-plot), we found 26 and 23 deregulated metabolites respectively (Supplemental Tables S3 and S4).

**Figure 2:**
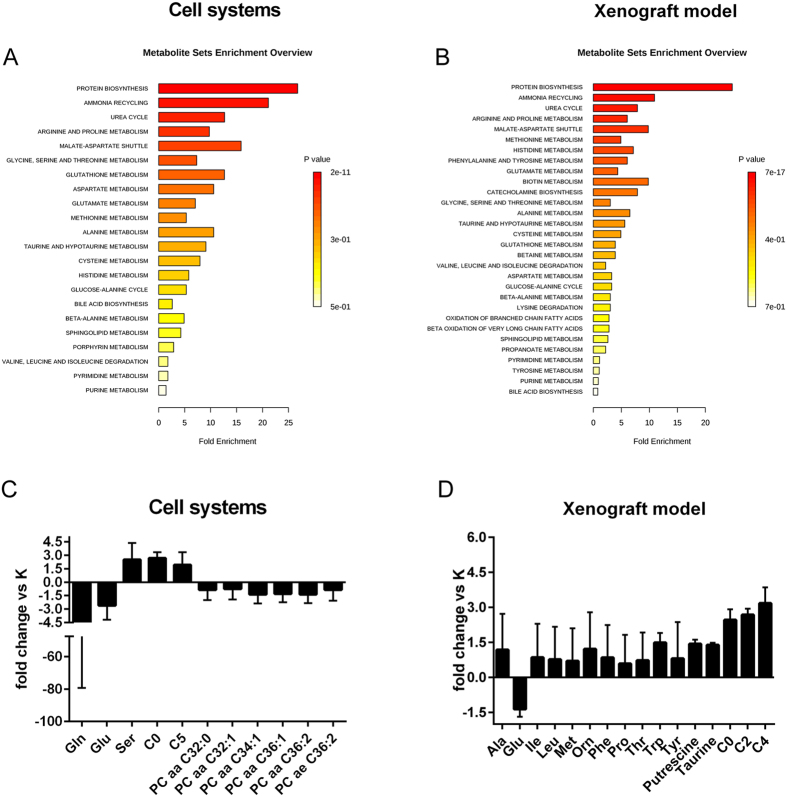
Metabolic pathway analysis highlighted the biochemical similarity between cell line and xenograft tumor. Metabolic pathway analyses related to the metabolites that significantly differ in G12C NSCLC KRAS mutant cell lines and their relative xenograft model compared to KRAS WT, utilizing the MetaboAnalyst functional interpretation tools. Panel A, graphic summary of metabolite set enrichment analysis for G12C KRAS cell line. Panel B, graphic summary of metabolite set enrichment analysis for G12C KRAS derived xenograft model. The horizontal bars summarize the main metabolite sets identified in this analysis; the bars are coloured based on their *p-values* and the length is based on the -fold enrichment. Panel C, histograms of significant difference (p < 0.05, Mann-Whitney-Wilcoxon test) in abundance in NSCLC cell lines (as fold change between mutant and WT) of the metabolite subset mapped into the first three top-score enrichment category (protein synthesis, ammonia recycling and urea cycle). Panel D, histograms of significant difference (p < 0.05, Mann-Whitney-Wilcoxon test) in abundance in NSCLC tumor xenografts (as fold change between mutant and WT) of the metabolite subset mapped into the first three top-score enrichment category (protein synthesis, ammonia recycling and urea cycle).

Pathway enrichment analysis using these deregulated metabolites showed that G12C KRAS mutation induced alteration of the same metabolic pathways both in cell monolayer systems and tumor xenograft models, supporting the evidence that metabolic rewiring induced by G12C KRAS mutation is also maintained *in vivo*. Particularly, protein biosynthesis, ammonia recycling and urea cycle were the most enriched pathways both in G12C KRAS mutant cell system and xenograft model (Fig. 2A,B). When we focused on deregulated metabolites whose abundances changed significantly (p < 0.05, Mann-Whitney-Wilcoxon test) between G12C and WT KRAS isoforms in both monolayer cell systems and in xenograft models, we found the significant alteration of 11 and 16 metabolites respectively (Fig. 2C,D). As we previously reported^1^ G12C KRAS mutation in cell systems lowered the concentrations of glutamine and glutamate, all involved in providing energy for growing and proliferation. In parallel, G12C KRAS mutation in the xenograft tumor lowered its glutamate abundance, indicating that in the whole tumor too, the G12C KRAS mutation used glutaminolysis as a source of energy to fuel tumor growth. Glutamate and glutamine are the two important amino acids in maintaining the nitrogen balance in the cell, supporting the central role of glutaminolysis and nitrogen anabolism to sustain cancer cell growth and proliferation in G12C KRAS mutant^7^.

The mutation in the xenograft tumor showed further metabolic adaptations to support tumor growth significantly increasing the consumption of many amino acids whose abundance did not change in the G12C KRAS cell system.

To note, G12C KRAS mutation in both cell and xenograft models induced a significant increase in the levels of carnitine, acetyl-carnitine and butyryl-carnitine (Fig. 2C,D). The enhanced concentrations of carnitine and its esters which are necessary intermediates in the oxidation of fatty acids^8^ could be associated with an enhanced mitochondrial fatty acids beta oxidation to support the increasing energy demand of the mutant KRAS to fuel again their growth and proliferation.

In contrast, only G12C KRAS cell mutants showed a significant (p < 0.05, Mann-Whitney-Wilcoxon test) down regulation of low-unsaturated very long-chain phosphatidylcholine (PC) species in comparison to WT KRAS (Fig. 2C). This lipophilic profile observed in G12C KRAS mutants could be associated with the recently highlighted ability of the G12C KRAS mutational status to induce micropinocytosis to promote engulfment of the nutrient from the extracellular environment to fuel cancer cell proliferation^9^. Alternatively, these changes in PC profile might be an important source of second messengers (i.e. phosphatidic acid) capable of acting on MAPK and PI3K/Akt signaling pathways^10^,^11^. To note, we did not observe any significant difference in the lipid profile of the xenograft models (Fig. 2D). Indeed the PCs, whose abundance was statistically different between KRAS isoforms in cell lines, had concentrations under our limit of quantitation in the xenograft model. Because of the robustness and reproducibility of the technology used we can exclude that the observed results might be due to analytical bias. Such discrepancy in the lipid profile deserved further attention in future studies.

Altogether, the data of our pilot investigation highlighted that key metabolic pathways involved in the metabolic rewiring induced by G12C KRAS mutation in cells growing in culture were also maintained in the derived xenograft model, suggesting that a more simple *in vitro* cell model can give important insights into the metabolic alterations induced by cancer cells to fuel their proliferation and survival. Our data also indicate that the gross changes observed *in vitro* are maintained in a more complex system of cancer growth *in vivo*. This implies that pharmacological modulation of cancer cell metabolism can be studied both *in vitro* and *in vivo* thus allowing a proper evaluation of the impact of new molecules acting at different steps in the cell metabolism. The possibility to evaluate the pharmacological (and toxicological) activity of new molecules and combination and to correlate their activity with the pharmacodynamic changes observed at metabolic level, will definitely help in defining the best rational way of use and combine these new molecules with potential activity as anticancer agents.

Nevertheless, caution has to be taken when translating tumor metabolism from *in vitro* to *in vivo* system due to tumor heterogeneity, tumor environment and KRAS mutational status able to affect metabolism as reported by two recent articles^12^,^13^ while our was under consideration.

## Material and Methods

### Cell cultures

Human non-small-cell lung carcinoma NCI-H1299 overexpressing G12C and WT KRAS isoforms were grown in RPMI1640 medium with 500 µG/mL of G418 (Gibco) added. G12C and WT KRAS clones generation and characterization are described in our previous articles^6^,^14^. Cells were maintained at 37 °C in a humidified atmosphere of 5% (v/v) CO_2_ in air^1^,^6^. Cells are routinely tested for mycoplasma contamination by PCR and authenticated with the PowerPlex 16 HS System (Promega) every 6 months by comparing the STR profiles to which deposited in ATCC and/or DSMZ databases.

### *In vivo* xenograft

Immunodeficient mice were obtained from Harlan-Italy and maintained under specific pathogen-free conditions with food and water provided ad libitum. Procedures involving animals and their care were conducted in conformity with the following laws, regulations, and policies governing the care and use of laboratory animals: Italian Governing Law (D.lgs 26/2014; Authorisation n.19/2008-A issued March 6, 2008 by Ministry of Health), Mario Negri Institutional Regulations and Policies providing internal authorisation for persons conducting animal experiments (Quality Management System Certificate – UNI EN ISO 9001:2008 – Reg. No. 8576-A), the NIH Guide for the Care and Use of Laboratory Animals (2011 edition), the EU directives and guidelines (EEC Council Directive 2010/63/UE) and in line with Guidelines for the welfare and use of animals in cancer research. Animal experiments has been reviewed and approved by the IRFMN Animal Care and Use Committee (IACUC) that includes members “ad hoc” for ethical issues. Animals were housed in the Institute’s Animal Care Facilities, which meet international standards; they are regularly checked by a certified veterinarian who is responsible for health monitoring, animal welfare supervision, experimental protocols and procedures revision.

Exponentially growing cells (both WT KRAS and G12C KRAS) were implanted subcutaneously (5 × 10^6^ cells/mouse) in the flank of immunodeficient mice (Harlan Laboratories). When the tumor reached approximately 500 mm^3^ as determined by caliper measurement of its diameters, tumors from 8 mice (4 bearing wt KRAS and 4 bearing G12C KRAS) were excised, and immediately frozen in liquid nitrogen and stored under cryogenic conditions until analysis. A portion of the tumor was used to determine the percentage of normal infiltrating cells (of murine origin) in the tumor (human origin) by species specific Real-Time PCR measurement of DNA.

### Real-Time PCR

Genomic DNA extracted with Maxwell 16 (Promega) was amplified by 7900HT Sequence Detection System (Life Technologies) with Primers and TaqMan probes specific for human and murine actin purchased as ready-to-use solutions (Life Technologies).

### Metabolomic sample preparation

NCI-H1299 cell lines harbouring G12C or WT KRAS isoforms were seeded at 75000 cells/mL in Petri dishes in a total volume of 10 mL in biological replicates (n = 9) for each clone. Forty-eight hours after seeding, all clones were in exponential growth with same proliferation rate. Metabolites were quenched and extracted as reported^1^. Briefly, NSCLC cells of each clone (average number of cells for WT KRAS: mean ± SD, 3.770.800 ± 72.200; for G12C KRAS: mean ± SD, 3.954.000 ± 10.1140) were rapidly rinsed in saline solution (~2 s), aspirated, and metabolism was quenched by adding ~15 mL of liquid N2 to the dish. The plates were then stored at −80 °C, and extracted and analyzed within seven days. Extraction was done by adding 1 mL of ice-cold MeOH to each plate and cells were scraped. Extracts were transferred to 1.5 mL micro-centrifuge tubes and pelleted at 4 °C for 15 min at 10000 × g. Supernatants were stored at −80 °C.

Frozen tumor tissue samples from each of the xenograft models (n = 4 for each clone) were disintegrated with a Mikro-Dismembrator S (Sartorius, Florence, Italy) at 3000 revolutions per minute for 40 s. The obtained powder was resuspended in ice-cold MeOH (3 µL/mG of tumor tissue) and homogenized for 1 min. Homogenized samples were subsequently centrifuged for 15 min at 10000 × g and supernatants stored at −80 °C. Thirty µL of each supernatant were used for targeted metabolomics analysis.

### Target metabolomics analysis

A targeted quantitative approach using a combined direct flow injection and liquid chromatography (LC) tandem mass spectrometry (MS/MS) assay (AbsoluteIDQ p180 kit, Biocrates) was applied for the metabolomics analysis. The assay quantifies 188 metabolites from five analyte groups: acylcarnitines, amino acids, biogenic amines, hexoses (sum of hexoses), PCs, and sphingomyelins (SMs) (Supplementary Table S5). The method combines derivatization and extraction of analytes with the selective mass-spectrometric detection using multiple reaction monitoring (MRM) pairs. Samples were analyzed (30 µ*L*) using an LC/MS (Triple quad 5500; AB Sciex) method (for analysis of amino acids and biogenic amines) followed by FIA-MS (analysis of lipids, acylcarnitines and hexose). For analytical specifications, refer to the AbsoluteIDQ p180 Kit manuals and Supplementary method section. The method of AbsoluteIDQ p180 kit has been proven to be in conformance with FDA Guideline ‘Guidance for Industry—Bioanalytical Method Validation, which implies proof of reproducibility within a given error range. Data evaluation, normalization (cell number or tumor weight) for quantification of metabolite concentrations and quality assessment have been performed with the MetIDQ software package, which is an integral part of the AbsoluteIDQ kit. The metabolite concentration of each metabolite in each experimental condition was compared with the measurement detection limit specifications as reported by the manufacturer of the AbsoluteIDQ p180 kit (Biocrates). A metabolite was excluded from further analyses if its concentration measurement data did not meet all of the following criteria: (1) minor of 20% of missing values (non-detectable peak) for each quantified metabolite in each experimental group (2) 50% of all measured sample concentrations for the metabolite had to be above the limit of detection (LOD).

### Multivariate data analysis

Metabolites concentrations (µM) from each experimental condition (cell clones and tumor tissues) were submitted to the SIMCA-P13 software package (Umetrics) for multivariate data analysis. Metabolite levels were Pareto scaled with mean centering and analyzed by orthogonal partial least-squares discriminant analysis (OPLS-DA) to maximize class discrimination. Loading S-plot generated by OPLS-DA were used to visualize the relationship between covariance and correlation within the OPLS-DA results. For the extraction of metabolites with highest magnitude and highest reliability in discriminating between groups, the cut-off of variable confidence was set at 0.05 for the increased metabolites and −0.05 for the decreased metabolites in the S-plot. The selected metabolites were then submitted to pathway analysis. Mann-Whitney-Wilcoxon test (JMP pro12, SAS Institute) was performed on the discriminant metabolites set to identify those whose concentration significantly (p < 0.05) changed between G12C and WT KRAS isoforms in cell and xenograft models.

### Metabolic pathway analysis

For biological interpretation of the metabolite dataset, we mapped the quantified metabolites to the KEGG pathway database (Kyoto Encyclopedia of Genes and Genomes; (www. genome.jp/kegg/), using MetaboAnalyst 3.0, a comprehensive online tool suite for metabolomic data analysis and interpretation (www.metaboanalyst.ca). Enrichment analysis (EA) tools were used to identify metabolic pathways that were most likely to be associated with the given list of the discriminant metabolites derived from the S-plots analysis.

## Additional Information

**How to cite this article**: Brunelli, L. *et al.* Comparative metabolomics profiling of isogenic KRAS wild type and mutant NSCLC cells in vitro and in vivo. *Sci. Rep.* **6**, 28398; doi: 10.1038/srep28398 (2016).

## Supplementary Material

Supplementary Information
