# Supplementary material for: Comparative metabolomics profiling of isogenic KRAS wild type and mutant NSCLC cells in vitro and in vivo
Source: Sci Rep. 2016 Jun 22;6:28398. doi: 10.1038/srep28398 (PMC4916601; doi:10.1038/srep28398)
Supplement: Supplementary Information [file srep28398-s1.pdf]

Comparative metabolomics profiling of isogenic KRAS wild type and mutant NSCLC cells in vitro and in vivo

Laura Brunelli[1*#], Elisa Caiola[2*#], Mirko Marabese[2], Massimo Broggini[2], Roberta Pastorelli[1]

[1]Protein and Gene Biomarkers Unit, Laboratory of Mass Spectrometry, Department of Environmental Health Sciences, IRCCS-Istituto di Ricerche Farmacologiche "Mario Negri", Milan, Italy.

[2]Laboratory of Molecular Pharmacology, Department of Oncology, IRCCS-Istituto di Ricerche Farmacologiche "Mario Negri", Milan, Italy

[#] These authors contributed equally to this work

***Corresponding authors: Laura Brunelli (laura.brunelli@marionegri.it) and Elisa Caiola (elisa.caiola@marionegri.it)

SUPPLEMENTARY INFORMATION

- Supplementary Methods
- Supplementary Figure S1: Tumor growth of G12C KRAS or WT KRAS cells in immunodeficient mice.
- Supplementary Table S1: Concentrations (uM) of all the metabolites measured in cell systems.
- Supplementary Table S2: Concentrations (uM) of all the metabolites measured in the xenograft model.
- Supplementary Table S3: Deregulated metabolites and their concentrations in cell systems.
- Supplementary Table S4: Deregulated metabolites and their concentrations in the xenograft model.
- Supplementary Table S5: List of the measurable metabolites using the Biocrates Absolute IDQ p180 kit.

Supplementary Methods

Absolute Metabolite profiling

Targeted metabolomics analysis of WT KRAS and G12C KRAS isoforms in NSCLC isogenic cell lines and their correspondent xenograft tumor samples was performed using the Biocrates AbsoluteIDQTM p180 kit (Biocrates Life Science AG, Innskruck, Austria). This validated targeted assay allows for simultaneous detection and quantification of metabolites in biological samples in a high-throughput manner. The metabolite extracts were processed following the instructions by the manufacturer and analyzed on a triple-quadropole mass spectrometer (AB SCIEX triple-quad 5500) operating in the multiple reaction monitoring (MRM-MS) mode. The assay is based on PITC (phenylisothiocyanate)-derivatization in the presence of internal standards for the analysis of aminoacids and biogenic amines resolved and quantified by liquid chromatography- tandem mass spectrometry (LC-MS/MS) using scheduled MRMs. Subsequent flow injection analysis tandem mass spectrometry (FIA-MS/MS) was performed to analyze acylcarnitines, glycerophospholipids, hexose. MRM detection was used for quantification applying spectra parsing algorithm integrated into the MetIQ software (Biocrates Life Science AG, Innskruck, Austria). Concentrations were calculated and evaluated by comparing measured analytes in a defined extracted ion count section to those of specific labeled internal standards or non-labeled ones, provided by the kit. Metabolite concentration were normalized based on cell numbers and tumor weight using cell normalization and tissue factor tools implemented into MetIDQ-boron software (Biocrates). The measurements are made in a 96-well format. Seven calibration standards, five quality control samples, three zero samples (methanol) and one blank (solvents) are integrated into the plate. The limit of detection for the individual metabolites is set three times the value of the “zero samples”. The average coefficient of variation of the metabolites among the biological replicates was 30%. This variation is the sum of biological and technical variation. Based on the five quality controls (QCs) included in the mass spectrometric analysis to monitor the instrumental performances and evaluate the quality of the

data, the CV was below 15% (technical variation). For glycerophospholipids, the precise position of the double bonds and the distribution of the carbon atoms in different fatty acid side chains cannot be determined with this technology. Lipid side-chain composition is abbreviated as Cx:y, where x denotes the number of carbons in the side chain and y the number of double bonds. The nature of fatty acids linkage is expressed as aa for diacyl or ae for acyl-alkyl. For example, PCaaC32:1 denotes diacyl-phosphatidylcholine with 32 carbons in the two fatty acids side chains and a single double bond in one of them. The list of all the measurable metabolites is provided in **supplementary table S5**.

Supplementary Figure 1. Tumor growth of G12C KRAS or WT KRAS cells in immunodeficient mice. Mice were inoculated subcutaneously with 200 uL of cell suspension containing $5x10^6$ WT or G12C KRAS cells. The growing tumor masses were measured with a Vernier caliper, and the tumor weights ($1mm^3$ = 1mg) were calculated with the formula: length x $(width)^2/2$. The tumor weight on day n was expressed as relative tumor weight (RTW) according to the following formula: RTW = TWn/TW0, where TWn is the tumor volume on day n and TW0 is the volume on the day of the first measurement.

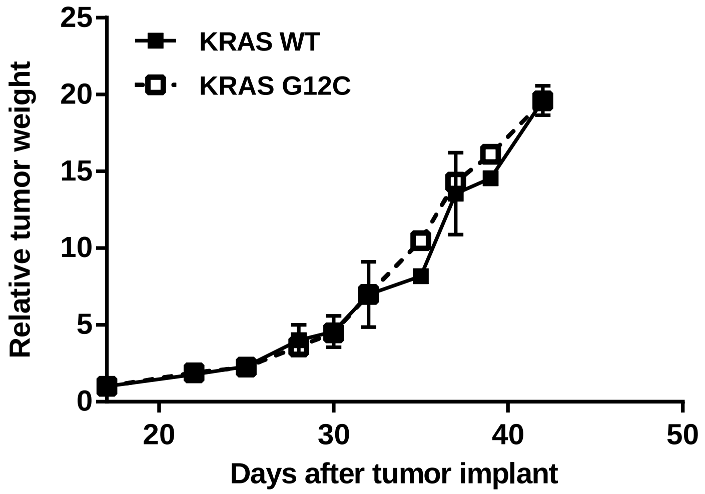

Supplementary Table S1. Micromolar concentrations of quantified metabolites in the monolayer cell system harboring G12C or WT KRAS isoforms. R# number of biological replicates

	WT									G12C								
Metabolite	R1	R2	R3	R4	R5	R6	R7	R8	R9	R1	R2	R3	R4	R5	R6	R7	R8	R9
lysoPC a C16:0	5.00	7.13	4.87	5.90	7.22	5.71	2.85	7.00	10.58	10.04	9.71	11.04	11.33	8.36	10.84	7.96	6.92	6.78
lysoPC a C16:1	0.95	1.22	0.90	0.81	1.11	0.96	0.54	1.24	1.87	1.71	1.38	1.16	1.71	1.35	1.72	2.21	1.68	1.68
lysoPC a C17:0	0.34	0.43	0.28	0.37	0.43	0.38	0.19	0.36	0.49	0.64	0.63	0.94	0.72	0.56	0.70	0.48	0.45	0.41
lysoPC a C18:0	2.09	2.88	2.05	2.07	2.72	2.45	0.93	1.78	2.88	4.47	4.07	9.90	3.51	3.70	4.10	1.96	1.70	1.61
lysoPC a C18:1	4.98	7.50	5.52	4.75	6.78	5.34	3.09	6.58	10.09	8.38	6.86	6.76	7.67	6.13	8.06	7.52	6.50	6.62
lysoPC a C18:2	0.36	0.44	0.35	0.32	0.44	0.34	0.23	0.39	0.49	0.57	0.42	0.54	0.48	0.47	0.51	0.60	0.47	0.48
lysoPC a C20:3	0.21	0.27	0.22	0.17	0.27	0.20	0.11	0.22	0.28	0.41	0.28	0.49	0.30	0.26	0.34	0.34	0.28	0.31
lysoPC a C20:4	0.17	0.40	0.34	0.19	0.36	0.23	0.09	0.28	0.38	0.57	0.38	0.60	0.31	0.23	0.37	0.36	0.20	0.25
lysoPC a C24:0	0.21	0.25	0.23	0.12	0.22	0.17	0.12	0.13	0.22	0.31	0.22	0.23	0.28	0.28	0.31	0.32	0.16	0.20
lysoPC a C26:0	0.61	0.61	0.67	0.26	0.60	0.41	0.52	0.36	0.53	0.96	0.69	0.57	0.67	0.75	0.93	1.00	0.46	0.55
lysoPC a C26:1	0.30	0.38	0.31	0.17	0.31	0.23	0.16	0.21	0.33	0.49	0.36	0.40	0.40	0.41	0.47	0.43	0.33	0.39
lysoPC a C28:0	0.72	1.01	0.75	0.46	0.89	0.75	0.50	0.52	0.95	1.35	1.03	0.91	0.87	1.17	1.26	1.35	1.15	1.19
lysoPC a C28:1	0.58	0.74	0.62	0.31	0.64	0.52	0.29	0.38	0.60	0.91	0.75	0.61	0.74	0.77	0.93	0.83	0.68	0.74
PC aa C24:0	0.25	0.26	0.29	0.12	0.23	0.19	0.17	0.13	0.16	0.25	0.16	0.14	0.19	0.16	0.25	0.32	0.12	0.15
PC aa C28:1	2.00	2.61	2.26	1.05	2.08	1.98	1.01	1.35	2.01	2.38	1.86	1.38	1.68	1.76	2.39	1.86	1.75	1.81
PC aa C30:0	11.17	15.50	8.42	6.06	11.49	11.75	6.18	9.54	14.23	13.07	11.35	9.23	8.57	9.72	11.38	9.39	10.43	11.52
PC aa C30:2	0.46	0.57	0.32	0.30	0.51	0.54	0.29	0.40	0.55	0.77	0.70	0.50	0.78	0.78	0.76	0.74	0.87	0.93
PC aa C32:0	134.84	181.53	105.09	51.56	105.07	115.46	66.88	111.75	129.60	124.10	104.89	77.96	61.02	78.78	89.70	68.81	71.06	77.84
PC aa C32:1	1212.67	1729.86	1037.92	527.04	1188.41	1274.96	634.76	1218.41	1597.02	1178.40	1058.83	796.41	772.73	941.13	1073.20	779.58	914.56	974.06
PC aa C32:2	157.59	226.03	139.44	83.52	162.73	172.21	88.29	185.96	238.64	168.34	155.29	103.00	134.90	161.09	172.85	151.78	197.92	192.06
PC aa C32:3	8.26	11.99	7.11	4.88	9.01	9.36	3.62	7.59	10.18	9.44	7.92	5.87	7.27	8.79	9.60	5.99	7.68	8.03
PC aa C34:1	2590.89	3280.84	1991.96	1041.31	2257.43	2346.00	1182.90	2162.59	2811.60	1732.02	1472.37	1410.90	1106.49	1380.30	1531.45	1074.04	1203.25	1255.24
PC aa C34:2	895.25	1357.15	760.92	387.52	842.86	932.75	454.38	950.86	1286.91	866.89	792.54	595.05	576.36	711.37	757.13	643.82	768.84	813.88
PC aa C34:3	56.51	82.74	48.50	27.41	57.55	60.19	25.52	56.90	78.19	67.35	59.43	45.31	47.31	55.38	62.72	43.79	57.78	57.67
PC aa C34:4	10.00	13.90	8.69	6.00	11.15	11.99	4.37	10.20	12.55	12.16	11.17	7.47	9.14	10.82	11.93	7.54	9.65	9.74

PC aa C36:0	12.73	21.06	10.86	5.10	12.81	15.57	5.65	10.83	16.56	13.23	12.44	9.54	7.70	10.66	11.00	7.59	8.68	8.45
PC aa C36:1	167.44	257.67	140.54	67.90	134.00	159.41	60.50	112.83	134.68	148.39	111.26	112.14	72.19	85.96	104.63	56.88	57.96	73.24
PC aa C36:2	1293.36	1961.16	1104.45	498.09	1104.33	1265.52	598.87	1077.50	1684.75	992.03	860.27	722.50	539.59	703.18	774.72	574.99	683.49	729.15
PC aa C36:3	189.48	297.10	158.58	78.45	178.66	194.90	77.14	164.84	233.97	202.95	167.66	139.76	112.51	140.33	151.77	102.36	124.23	128.94
PC aa C36:4	78.45	119.80	66.80	36.57	84.39	93.65	30.21	61.05	85.00	100.40	89.32	68.71	57.13	73.62	79.76	42.42	52.17	53.83
PC aa C36:5	31.85	48.07	28.40	17.20	37.40	39.73	12.97	26.92	34.99	46.44	40.57	30.84	28.55	35.15	38.13	19.67	24.48	24.36
PC aa C36:6	16.59	25.19	13.29	7.11	15.82	17.94	5.87	13.98	19.68	15.47	12.42	10.28	9.25	11.36	12.87	8.01	9.65	9.89
PC aa C38:0	8.68	14.57	7.70	3.69	8.74	8.95	2.95	6.09	8.79	10.99	8.78	8.16	5.70	7.81	8.20	5.89	6.76	7.15
PC aa C38:3	25.57	40.02	20.63	10.30	22.57	23.95	9.89	17.17	25.96	28.18	21.83	21.36	14.09	18.05	20.52	12.92	13.53	14.57
PC aa C38:4	42.03	67.97	33.98	17.42	40.71	44.16	13.35	26.54	38.41	53.37	43.73	38.13	26.68	36.13	37.84	21.66	24.97	25.73
PC aa C38:5	59.10	90.63	52.75	28.41	62.80	67.88	23.16	41.49	61.56	71.35	58.60	51.21	41.07	51.91	53.16	30.77	35.90	37.03
PC aa C38:6	53.70	78.61	43.87	25.41	55.88	60.12	17.40	34.88	50.31	61.22	53.75	42.37	34.61	44.49	48.15	25.17	28.96	31.77
PC aa C40:2	2.66	4.34	2.36	1.16	2.26	2.64	1.64	2.63	3.40	2.35	2.31	2.09	1.58	2.21	2.28	1.77	1.50	2.33
PC aa C40:3	2.35	3.63	1.97	0.84	1.89	2.13	1.13	1.97	2.38	2.29	1.75	2.11	1.28	1.72	1.79	1.38	1.21	1.58
PC aa C40:4	3.47	6.34	3.51	1.92	4.17	3.87	1.62	2.78	3.66	4.23	3.40	3.36	2.31	3.05	3.30	2.01	2.20	2.50
PC aa C40:5	14.42	22.47	11.32	6.37	14.02	15.65	5.38	9.22	14.23	14.81	12.09	11.49	8.30	9.95	10.64	5.90	6.51	7.59
PC aa C40:6	20.50	33.22	19.83	10.00	21.87	23.10	7.20	14.01	21.30	26.81	22.54	19.08	13.75	18.96	20.45	11.17	12.47	12.44
PC aa C42:1	0.86	0.85	0.52	0.41	0.58	0.62	0.46	0.60	0.75	0.83	0.70	0.49	0.52	0.72	0.60	0.80	0.70	0.80
PC aa C42:2	0.63	1.26	0.75	0.41	0.55	0.76	0.56	0.83	1.15	1.16	0.82	0.89	0.69	1.00	0.90	1.06	0.82	1.08
PC aa C42:4	0.70	1.10	0.72	0.47	0.85	0.59	0.38	0.78	0.79	0.81	0.63	0.66	0.52	0.59	0.72	0.45	0.47	0.57
PC aa C42:5	2.38	3.18	1.77	0.98	2.13	2.39	0.83	1.26	2.04	2.31	1.73	1.72	1.24	1.67	1.60	0.99	0.92	1.11
PC aa C42:6	5.39	9.79	5.22	2.97	6.37	6.49	2.19	4.31	6.88	5.80	4.65	4.45	3.28	3.85	4.60	2.70	2.63	3.16
PC ae C30:0	2.41	3.30	1.87	1.18	2.30	2.38	1.36	2.10	3.11	3.65	3.00	2.55	2.29	2.54	3.03	2.87	3.28	3.36
PC ae C30:1	1.22	1.90	1.02	0.63	1.15	1.26	0.59	0.88	1.25	1.67	1.49	1.22	1.23	1.23	1.54	1.23	1.46	1.62
PC ae C30:2	0.18	0.25	0.15	0.12	0.19	0.20	0.09	0.15	0.19	0.25	0.25	0.17	0.22	0.23	0.25	0.18	0.20	0.21
PC ae C32:1	204.96	289.80	174.19	76.18	176.72	189.69	98.01	188.44	249.53	226.65	197.21	147.37	134.42	165.83	192.67	163.59	192.11	203.75
PC ae C32:2	47.37	68.87	40.13	20.63	41.24	44.80	20.56	40.41	50.74	42.34	37.44	30.22	27.23	33.25	37.77	25.55	31.85	34.22
PC ae C34:0	31.01	37.97	22.23	11.68	24.87	22.97	14.82	27.21	29.32	33.40	26.37	19.94	19.41	24.20	25.87	19.94	19.86	22.59
PC ae C34:1	453.40	662.23	374.08	170.96	363.86	431.20	211.74	389.27	533.38	419.26	365.18	299.45	232.72	281.55	321.64	253.57	291.30	306.12
PC ae C34:2	182.06	269.49	150.77	75.69	151.28	174.19	80.55	149.08	199.30	165.66	139.14	112.66	96.56	114.55	131.18	98.61	117.69	126.21

PC ae C34:3	24.25	36.52	20.07	10.54	22.81	24.56	9.84	21.06	29.12	21.12	18.68	15.27	13.08	16.01	18.43	12.47	14.88	15.99
PC ae C36:0	11.63	17.89	9.66	6.34	12.24	14.04	6.30	14.34	17.87	10.22	8.65	7.26	7.37	9.09	9.39	7.05	8.75	8.15
PC ae C36:1	94.13	138.17	79.35	39.22	81.65	89.92	43.42	72.06	94.59	90.60	74.38	63.57	48.00	59.91	69.54	47.94	53.23	56.98
PC ae C36:2	178.93	262.83	145.60	70.97	155.18	173.76	78.79	143.75	212.80	160.17	136.69	118.04	92.07	115.11	125.28	92.28	107.70	118.49
PC ae C36:3	71.64	116.22	59.72	29.68	63.99	71.60	31.43	67.68	103.73	68.79	56.00	49.95	37.37	48.70	52.83	36.91	46.57	47.54
PC ae C36:4	45.15	66.16	38.12	18.69	41.56	44.76	17.60	34.13	50.93	55.08	47.80	37.38	31.17	37.15	42.63	28.39	33.87	36.08
PC ae C36:5	27.17	40.68	23.36	11.93	25.20	28.36	10.04	20.04	28.21	35.73	30.86	23.67	20.70	25.47	28.60	17.86	22.55	22.26
PC ae C38:0	9.10	14.50	8.01	5.07	10.44	10.85	3.95	8.13	11.44	11.06	9.44	6.99	7.62	9.03	9.52	6.52	8.37	8.33
PC ae C38:1	5.82	8.94	5.38	2.40	5.17	5.93	2.62	4.49	6.36	5.57	4.96	5.02	3.61	4.49	4.82	4.24	3.39	4.15
PC ae C38:2	28.81	44.28	25.58	13.13	25.58	29.54	14.45	24.02	31.90	26.77	22.86	21.65	15.13	19.31	21.32	16.53	17.69	19.38
PC ae C38:3	20.85	35.61	17.11	7.72	17.72	20.31	8.68	17.29	24.62	20.02	15.65	14.99	9.82	13.58	15.01	9.39	10.14	11.63
PC ae C38:4	22.22	37.23	20.29	9.54	20.87	22.44	7.84	15.74	24.91	27.24	21.40	20.05	14.04	17.61	19.15	13.04	14.98	16.24
PC ae C38:5	34.63	55.83	29.60	13.36	30.58	34.64	13.11	25.34	36.44	44.70	36.49	30.53	23.55	28.55	33.86	22.55	27.12	29.16
PC ae C38:6	32.81	52.35	26.94	12.70	29.57	33.70	10.58	22.78	33.02	41.51	33.93	27.86	23.51	27.87	31.90	20.46	24.65	26.24
PC ae C40:1	2.04	2.96	1.74	1.12	2.00	1.85	0.66	1.48	1.88	2.49	2.23	1.59	1.64	1.91	1.96	1.24	1.59	1.47
PC ae C40:2	5.89	7.77	5.30	3.19	5.20	5.06	3.49	5.86	6.17	4.32	3.59	3.86	2.92	3.52	3.46	2.82	3.07	3.20
PC ae C40:3	3.00	4.31	2.96	1.38	2.68	3.02	1.58	2.72	3.08	2.98	2.70	2.66	1.85	2.29	2.52	1.89	1.93	1.82
PC ae C40:4	4.04	5.93	3.19	1.82	3.86	4.07	1.65	2.86	3.83	4.45	3.80	3.49	2.62	3.47	3.56	2.75	2.84	3.36
PC ae C40:5	12.53	19.80	10.53	5.12	11.63	12.93	5.17	8.51	13.97	15.17	12.49	10.95	8.15	10.31	11.46	8.01	8.40	9.48
PC ae C40:6	10.70	16.71	9.07	4.60	9.77	10.90	3.76	7.48	10.84	13.74	10.97	10.15	7.30	8.95	10.35	7.00	7.82	8.29
PC ae C42:0	2.50	3.42	2.04	1.31	2.41	2.32	1.08	1.64	2.54	2.56	2.42	1.98	1.62	2.02	1.96	1.31	1.48	1.52
PC ae C42:2	1.25	1.41	0.86	0.62	1.05	1.06	0.40	0.65	0.84	1.27	1.05	1.01	0.78	0.78	0.89	0.74	0.56	0.75
PC ae C42:3	1.32	1.78	1.09	0.71	1.27	1.20	0.67	0.91	1.43	1.53	1.18	1.34	0.90	1.21	1.07	0.73	0.86	0.81
PC ae C42:4	1.11	1.49	0.64	0.58	1.04	1.13	0.50	0.99	1.02	1.11	1.00	1.09	0.67	1.11	0.92	0.61	0.65	0.72
PC ae C42:5	2.46	3.78	2.29	1.49	2.26	2.40	1.28	2.15	2.46	2.69	2.18	2.08	1.50	2.00	2.06	1.54	1.44	1.92
PC ae C44:3	0.58	0.72	0.56	0.24	0.46	0.41	0.38	0.39	0.64	0.63	0.50	0.45	0.40	0.56	0.48	0.34	0.38	0.43
PC ae C44:4	0.49	0.68	0.49	0.31	0.51	0.57	0.32	0.50	0.50	0.52	0.40	0.37	0.28	0.46	0.37	0.24	0.26	0.27
PC ae C44:6	0.58	0.87	0.53	0.32	0.66	0.65	0.36	0.61	0.88	0.71	0.59	0.50	0.42	0.51	0.57	0.45	0.47	0.48
Sugar	3453.91	2795.92	1790.71	4281.02	3038.59	2997.45	1805.38	2700.42	4305.21	2052.24	2023.16	2137.81	5304.41	3895.19	3755.51	3203.01	4279.05	3689.19
Ala	271.00	270.67	189.33	353.33	380.00	386.67	810.00	1423.33	1520.00	793.33	693.33	109.67	1466.67	1123.33	1350.00	4166.67	4766.67	4466.67

Arg	114.00	98.00	70.00	147.67	126.00	107.67	179.67	228.67	269.33	111.67	113.00	92.33	167.00	165.33	132.00	300.00	293.67	298.00
Asn	653.33	660.00	433.33	790.00	843.33	846.67	1350.00	1903.33	1856.67	703.33	550.00	326.33	980.00	886.67	1183.33	1993.33	2136.67	2083.33
Asp	143.33	156.00	103.33	164.67	181.00	172.67	128.00	216.33	228.00	207.00	186.67	120.33	253.33	239.33	272.00	71.33	63.33	51.67
Cit	4.63	4.23	3.33	4.67	5.50	5.90	10.07	19.23	20.20	3.90	3.17	3.00	7.10	6.00	6.73	22.27	21.23	22.20
Gln	1056.67	1106.67	646.67	1136.67	1430.00	1076.67	1100.00	1680.00	1973.33	526.67	456.67	620.00	556.67	526.67	490.00	21.57	19.20	15.13
Glu	3866.67	3866.67	2806.67	4133.33	5166.67	4833.33	2653.33	4800.00	4366.67	2866.67	2406.67	1220.00	3566.67	2810.00	3600.00	730.00	970.00	843.33
Gly	546.67	540.00	327.00	543.33	616.67	536.67	550.00	913.33	1010.00	366.67	296.67	120.00	483.33	383.33	440.00	970.00	860.00	990.00
His	35.00	33.67	20.63	41.33	44.00	36.67	70.67	114.33	131.00	33.23	29.73	16.70	53.67	43.67	45.00	142.33	132.67	155.33
Ile	117.33	107.00	69.33	145.00	141.33	123.67	206.67	301.33	373.33	129.00	108.67	62.33	211.00	178.00	178.00	463.33	463.33	503.33
Leu	109.33	99.67	59.67	136.33	135.67	119.00	242.67	304.33	366.67	119.33	102.00	82.67	217.33	183.67	174.00	446.67	393.33	453.33
Lys	10.03	7.20	5.67	15.03	9.63	8.90	9.83	21.10	21.83	9.37	8.10	18.33	16.27	14.77	8.23	26.53	19.50	28.00
Met	23.87	26.83	16.73	31.27	33.30	27.97	40.33	60.00	78.33	23.57	22.37	16.47	36.67	33.67	34.33	75.67	75.00	82.00
Orn	5.23	4.27	3.05	10.83	8.43	6.47	13.70	31.27	32.13	5.63	4.80	3.28	11.70	10.63	7.83	46.67	31.80	43.00
Phe	27.43	25.10	16.53	32.87	32.33	30.27	38.33	54.00	70.00	25.97	22.93	17.33	41.00	35.00	34.67	69.33	71.00	75.33
Pro	275.00	278.67	173.33	309.33	353.33	291.33	523.33	580.00	616.67	270.33	244.67	103.67	380.00	363.33	370.00	570.00	503.33	566.67
Ser	64.67	60.67	36.00	64.33	74.33	68.67	10.50	17.73	13.97	83.00	71.33	82.67	90.00	84.33	81.00	62.33	53.33	72.67
Thr	90.67	90.67	58.67	117.00	119.67	130.67	195.33	312.33	325.00	102.33	80.33	50.33	154.33	126.33	154.67	350.00	436.67	390.00
Trp	8.87	8.23	5.67	10.43	10.60	9.33	13.37	18.60	24.23	8.83	7.53	5.47	13.70	11.47	11.47	26.87	27.17	28.67
Tyr	40.33	40.33	24.77	50.33	50.33	46.00	77.33	112.67	142.67	41.00	35.33	22.50	64.33	53.33	55.33	154.33	156.33	169.00
Val	31.40	31.00	19.23	40.00	38.33	36.33	43.00	60.00	79.33	33.67	28.80	25.17	48.67	42.67	42.67	83.00	81.00	89.67
Ac-Orn	1.16	1.06	0.82	1.63	1.59	1.37	3.47	7.83	9.13	1.40	1.08	0.39	2.17	1.77	1.91	16.30	8.50	12.37
ADMA	0.17	0.07	0.11	0.17	0.19	0.18	0.35	0.54	0.65	0.11	0.19	0.12	0.26	0.61	0.24	0.85	0.97	0.54
alpha-AAA	2.26	2.58	1.29	2.76	3.47	2.20	2.44	6.87	8.30	2.57	1.69	0.92	3.25	2.55	2.72	3.47	2.74	2.40
Carnosine	0.56	0.53	0.32	0.66	0.49	0.50	0.35	0.52	0.81	0.25	0.33	0.36	0.68	0.57	0.38	0.34	0.52	0.44
Met-SO	1.74	1.37	0.99	1.92	1.63	1.69	3.32	4.43	6.30	1.33	1.17	0.69	1.89	1.87	2.33	5.77	5.10	5.97
Putrescine	27.67	30.10	19.97	41.00	27.60	27.23	28.77	50.33	57.00	25.57	19.67	24.20	38.67	36.67	27.93	37.67	39.67	35.00
Serotonin	0.13	0.15	0.09	0.20	0.19	0.17	0.09	0.12	0.16	0.08	0.05	0.12	0.11	0.07	0.06	0.09	0.11	0.09
Spermidine	6.57	6.57	5.13	7.33	4.90	8.50	10.40	11.67	10.40	6.43	4.27	3.47	9.37	8.40	7.43	10.40	11.27	9.33
Spermine	3.33	4.10	3.57	5.13	3.43	7.57	9.70	8.87	6.50	3.26	2.39	1.35	5.83	4.23	4.73	6.63	8.33	5.63
t4-OH-Pro	229.67	223.00	138.33	263.00	286.33	249.67	576.67	713.33	900.00	243.67	227.00	101.33	426.67	353.33	396.67	1093.33	1080.00	1180.00

Taurine	204.67	202.00	158.67	222.33	229.33	218.67	188.33	221.33	217.00	216.33	204.67	119.33	267.33	251.33	257.00	259.33	254.33	234.33
total DMA	0.17	0.14	0.09	0.22	0.21	0.18	0.38	0.72	0.81	0.15	0.12	0.09	0.32	0.25	0.22	0.71	1.01	0.88
SM (OH) C14:1	1.49	2.06	1.20	0.93	1.82	1.54	0.89	1.37	2.17	2.16	1.89	1.69	1.61	2.05	2.14	1.57	1.76	1.90
SM (OH) C16:1	0.23	0.37	0.19	0.20	0.39	0.27	0.13	0.22	0.32	0.39	0.36	0.28	0.28	0.25	0.41	0.28	0.27	0.23
SM (OH) C22:1	0.10	0.11	0.03	0.02	0.09	0.02	0.03	0.07	0.18	0.20	0.14	0.10	0.09	0.11	0.10	0.16	0.13	0.13
SM (OH) C22:2	0.01	0.05	0.02	0.05	0.03	0.03	0.02	0.02	0.00	0.07	0.12	0.12	0.12	0.12	0.10	0.14	0.14	0.17
SM C16:0	22.36	32.34	17.10	12.78	23.96	22.24	11.07	17.89	28.04	24.50	20.69	19.30	16.75	19.78	22.81	15.35	17.32	19.54
SM C16:1	3.11	4.78	2.83	2.28	3.90	3.55	1.69	3.00	5.16	4.27	3.84	3.13	3.47	4.15	4.39	3.25	3.60	3.80
SM C18:0	0.30	0.44	0.20	0.17	0.19	0.34	0.06	0.03	0.08	0.27	0.35	0.38	0.17	0.05	0.17	0.05	0.05	0.05
SM C18:1	0.52	0.82	0.45	0.39	0.63	0.56	0.33	0.58	0.88	0.75	0.64	0.75	0.61	0.77	0.66	0.64	0.72	0.76
SM C20:2	0.14	0.28	0.10	0.10	0.18	0.14	0.07	0.16	0.31	0.20	0.14	0.22	0.13	0.22	0.10	0.16	0.25	0.23
SM C22:3	0.06	0.10	0.06	0.08	0.03	0.08	0.04	0.02	0.14	0.06	0.05	0.10	0.06	0.17	0.00	0.14	0.08	0.11
SM C24:0	1.37	2.29	1.09	0.80	1.38	1.39	0.88	1.28	2.31	1.99	1.51	1.99	1.32	1.67	1.60	1.65	1.72	2.06
SM C24:1	0.44	0.54	0.37	0.36	0.43	0.44	0.36	0.43	0.65	0.70	0.59	0.77	0.51	0.71	0.57	0.79	0.77	0.96
SM C26:0	0.03	0.05	0.02	0.02	0.02	0.02	0.01	0.02	0.02	0.04	0.01	0.03	0.02	0.01	0.01	0.03	0.05	0.03
SM C26:1	0.05	0.07	0.03	0.05	0.06	0.04	0.03	0.04	0.09	0.07	0.03	0.08	0.05	0.06	0.05	0.07	0.09	0.06
C0	6.59	7.04	4.22	6.63	7.47	6.53	6.22	7.44	10.10	14.44	12.70	9.15	23.46	18.46	20.18	22.50	23.29	21.81
C18:1	0.09	0.08	0.05	0.10	0.10	0.10	0.04	0.05	0.05	0.09	0.08	0.16	0.12	0.11	0.12	0.08	0.10	0.09
C2	2.88	2.76	1.69	3.51	3.70	3.13	4.17	5.52	6.59	2.86	2.77	1.19	5.13	4.53	4.68	15.98	17.66	18.38
C3	0.51	0.49	0.32	0.58	0.57	0.53	0.46	0.69	0.90	0.87	0.75	0.29	1.37	1.23	1.32	1.83	2.00	1.73
C3-DC-M / C5-OH	0.04	0.04	0.03	0.04	0.04	0.03	0.03	0.05	0.05	0.04	0.04	0.03	0.06	0.05	0.05	0.07	0.07	0.07
C4	3.38	3.20	1.89	3.67	3.68	3.34	3.43	4.94	6.54	6.83	6.08	2.98	11.25	9.06	9.57	12.39	12.90	12.43
C5	12.07	11.62	7.29	12.72	12.98	12.26	9.58	12.53	16.48	15.25	13.10	7.22	29.19	23.14	25.93	37.32	41.86	39.99

Supplementary Table S2. Micromolar concentrations of quantified metabolites in the xenograft tumor models harboring G12C or WT KRAS isoforms. R# number of biological replicates

Metabolites	WT				G12C			
	R1	R2	R3	R4	R1	R2	R3	R4
lysoPC a C16:0	0.333	0.347	0.255	0.682	1.47	0.667	1.21	0.883
lysoPC a C18:1	0.18	0.196	0.087	0.323	0.566	0.221	0.407	0.333
lysoPC a C18:2	0.193	0.216	0.123	0.488	0.663	0.359	0.575	0.492
lysoPC a C20:4	0.026	0.096	0.032	0.105	0.192	0.064	0.12	0.069
PC aa C32:0	0.53	0.184	0.382	0.082	0.599	1.06	0.174	0.135
PC aa C32:1	0.554	0.196	0.254	0.131	0.349	0.456	0.111	0.086
PC aa C34:1	2.19	0.772	1.29	0.251	1.47	2.86	0.372	0.342
PC aa C34:2	0.923	0.315	0.495	0.258	0.731	1.57	0.32	0.235
PC aa C36:1	0.297	0.144	0.224	0.049	0.2	0.442	0.057	0.056
PC aa C36:2	0.884	0.247	0.498	0.128	0.453	1.31	0.151	0.164
PC aa C36:3	0.377	0.14	0.226	0.16	0.331	0.687	0.155	0.112
PC aa C36:4	0.129	0.047	0.102	0.074	0.154	0.349	0.12	0.084
PC aa C38:3	0.098	0.037	0.082	0.015	0.063	0.187	0.028	0.021
PC aa C38:4	0.097	0.038	0.096	0.042	0.1	0.297	0.047	0.036
PC ae C34:2	0.119	0.044	0.08	0.026	0.052	0.162	0.021	0.019
sugars	95.9	17.4	88.7	101	74.7	49.4	41.6	112
Ala	550	314	231	317	523	463	560	600
Arg	29.9	14.5	19.6	19	22.8	20.5	27.7	21.8
Asn	60.7	48	40	63	42.3	56.7	47.3	55.7
Asp	79	46	37	79	49.3	45	69	75.7
Cit	45	40	34.7	35.3	30.5	29.8	37	37
Gln	203	92.7	115	133	62	106	81.3	96.3
Glu	1013	843	597	793	593	540	580	703
Gly	617	322	255	423	353	470	470	306
His	49.7	32.2	28.9	42	39.3	38.7	46.7	39.7
Ile	41.7	20.1	18.8	28.2	32.9	32.1	34.7	35.3
Leu	98.7	46.7	49	67.3	75	68	80.7	87.3
Lys	84.3	62	65.3	58.7	72.7	76.7	107	58.7
Met	59.3	30.3	28.5	40.3	43.7	34	49.7	53
Orn	11.5	9.6	9.37	10.4	11.2	21.4	22.4	13.2
Phe	56	26.8	31.9	37.7	45.7	43	53	51.7
Pro	224	131	130	149	178	143	171	180
Ser	63.3	37.3	29.9	38.3	41.7	38	70.3	57.7
Thr	176	152	127	155	168	204	191	173
Trp	13.7	8.53	9.67	12.3	14.7	14.8	18.8	14.8
Tyr	72.3	27.7	35.3	47.7	48	52	52.7	66
Val	70.7	50.3	40.3	69	75	66.7	93.7	69.7
Ac-Orn	2.62	1.41	1.68	1.79	1.5	1.14	2.02	1.56
ADMA	0.66	0.367	0.353	0.297	0.483	0.757	0.723	1.15
alpha-AAA	2.66	1.4	1.22	3.09	1.83	3.17	1.96	1.53
Carnosine	0.245	0.05	0.049	0.05	0.05	0.057	0.054	0.082
Creatinine	2.5	2.32	2.41	2.58	2.69	2.61	2.53	2.75
DOPA	0.184	0.114	0.133	0.151	0.157	0.204	0.238	0.222

Histamine	0.557	0.311	1.07	2.86	0.693	1.52	0.45	1.61
Kynurenine	0.52	0.279	0.329	0.423	0.186	0.287	0.312	0.264
Met-SO	2.21	1.22	0.947	1.84	1.32	0.997	1.9	1.44
Putrescine	3.67	2.73	2.73	2.34	4.73	4.63	3.73	3.27
Serotonin	0.023	0.019	0.024	0.061	0.028	0.049	0.023	0.028
Spermidine	15.5	9.8	12.8	11.8	14.2	9.53	13.2	11.6
Spermine	4.33	3.37	3.53	3.67	3.9	3.27	3.37	3.25
t4-OH-Pro	16	10.3	8.97	11.7	15.2	12.7	12.3	12.1
Taurine	160	159	160	138	244	209	220	186
total DMA	0.487	0.333	0.347	0.41	0.35	0.477	0.593	0.507
SM C16:0	0.369	0.126	0.21	0.085	0.255	0.494	0.101	0.088
SM C24:1	0.043	0.016	0.029	0.013	0.034	0.057	0.02	0.02
C0	3.19	2.6	3.88	3.39	9.75	6.69	8.12	7.19
C16	0.06	0.066	0.067	0.09	0.289	0.137	0.162	0.147
C16:1	0.035	0.026	0.025	0.036	0.107	0.06	0.053	0.044
C18:1	0.092	0.076	0.052	0.133	0.354	0.176	0.194	0.19
C18:2	0.02	0.024	0.019	0.039	0.152	0.109	0.098	0.098
C2	3.54	2.47	3.04	2.83	9.44	7.48	8.01	6.8
C3	0.072	0.072	0.066	0.079	0.178	0.15	0.2	0.162
C4	0.388	0.355	0.356	0.304	1.15	0.964	1.49	0.866
C3-DC (C4-OH)	0.07	0.05	0.067	0.049	0.115	0.101	0.104	0.087
C5	0.146	0.101	0.123	0.131	0.351	0.325	0.465	0.308

Supplementary Table S3. Metabolite concentrations (uM) and relative difference in abundance as fold change in the discriminant (from S-plots generated by OPLS-DA) metabolite between G12C and WT KRAS isoforms on monolayer cell system. R# number of biological replicates

Metabolites	WT									G12C									**Fold change G12C vs WT**
	R1	R2	R3	R4	R5	R6	R7	R8	R9	R1	R2	R3	R4	R5	R6	R7	R8	R9	
Sugar	3453.91	2795.92	1790.71	4281.02	3038.59	2997.45	1805.38	2700.42	4305.21	2052.24	2023.16	2137.81	5304.41	3895.19	3755.51	3203.01	4279.05	3689.19	1.12
Ala	271.00	270.67	189.33	353.33	380.00	386.67	810.00	1423.33	1520.00	793.33	693.33	109.67	1466.67	1123.33	1350.00	4166.67	4766.67	4466.67	3.38
Asn	653.33	660.00	433.33	790.00	843.33	846.67	1350.00	1903.33	1856.67	703.33	550.00	326.33	980.00	886.67	1183.33	1993.33	2136.67	2083.33	1.16
Gln	1056.67	1106.67	646.67	1136.67	1430.00	1076.67	1100.00	1680.00	1973.33	526.67	456.67	620.00	556.67	526.67	490.00	21.57	19.20	15.13	-3.47
Glu	3866.67	3866.67	2806.67	4133.33	5166.67	4833.33	2653.33	4800.00	4366.67	2866.67	2406.67	1220.00	3566.67	2810.00	3600.00	730.00	970.00	843.33	-1.92
Gly	546.67	540.00	327.00	543.33	616.67	536.67	550.00	913.33	1010.00	366.67	296.67	120.00	483.33	383.33	440.00	970.00	860.00	990.00	-1.14
Ile	117.33	107.00	69.33	145.00	141.33	123.67	206.67	301.33	373.33	129.00	108.67	62.33	211.00	178.00	178.00	463.33	463.33	503.33	1.45
Leu	109.33	99.67	59.67	136.33	135.67	119.00	242.67	304.33	366.67	119.33	102.00	82.67	217.33	183.67	174.00	446.67	393.33	453.33	1.38
Ser	64.67	60.67	36.00	64.33	74.33	68.67	10.50	17.73	13.97	83.00	71.33	82.67	90.00	84.33	81.00	62.33	53.33	72.67	1.66
Spermidine	6.57	6.57	5.13	7.33	4.90	8.50	10.40	11.67	10.40	6.43	4.27	3.47	9.37	8.40	7.43	10.40	11.27	9.33	-1.02
t4-OH-Pro	229.67	223.00	138.33	263.00	286.33	249.67	576.67	713.33	900.00	243.67	227.00	101.33	426.67	353.33	396.67	1093.33	1080.00	1180.00	1.43
Taurine	204.67	202.00	158.67	222.33	229.33	218.67	188.33	221.33	217.00	216.33	204.67	119.33	267.33	251.33	257.00	259.33	254.33	234.33	1.11
C0	6.59	7.04	4.22	6.63	7.47	6.53	6.22	7.44	10.10	14.44	12.70	9.15	23.46	18.46	20.18	22.50	23.29	21.81	2.67
C5	12.07	11.62	7.29	12.72	12.98	12.26	9.58	12.53	16.48	15.25	13.10	7.22	29.19	23.14	25.93	37.32	41.86	39.99	2.17
PC aa C32:0	134.84	181.53	105.09	51.56	105.07	115.46	66.88	111.75	129.60	124.10	104.89	77.96	61.02	78.78	89.70	68.81	71.06	77.84	-1.33
PC aa C32:1	1212.67	1729.86	1037.92	527.04	1188.41	1274.96	634.76	1218.41	1597.02	1178.40	1058.83	796.41	772.73	941.13	1073.20	779.58	914.56	974.06	-1.23
PC aa C34:1	2590.89	3280.84	1991.96	1041.31	2257.43	2346.00	1182.90	2162.59	2811.60	1732.02	1472.37	1410.90	1106.49	1380.30	1531.45	1074.04	1203.25	1255.24	-1.62
PC aa C34:2	895.25	1357.15	760.92	387.52	842.86	932.75	454.38	950.86	1286.91	866.89	792.54	595.05	576.36	711.37	757.13	643.82	768.84	813.88	-1.21
PC aa C36:1	167.44	257.67	140.54	67.90	134.00	159.41	60.50	112.83	134.68	148.39	111.26	112.14	72.19	85.96	104.63	56.88	57.96	73.24	-1.50
PC aa C36:2	1293.36	1961.16	1104.45	498.09	1104.33	1265.52	598.87	1077.50	1684.75	992.03	860.27	722.50	539.59	703.18	774.72	574.99	683.49	729.15	-1.61
PC aa C36:3	189.48	297.10	158.58	78.45	178.66	194.90	77.14	164.84	233.97	202.95	167.66	139.76	112.51	140.33	151.77	102.36	124.23	128.94	-1.24
PC ae C34:1	453.40	662.23	374.08	170.96	363.86	431.20	211.74	389.27	533.38	419.26	365.18	299.45	232.72	281.55	321.64	253.57	291.30	306.12	-1.30
PC ae C34:2	182.06	269.49	150.77	75.69	151.28	174.19	80.55	149.08	199.30	165.66	139.14	112.66	96.56	114.55	131.18	98.61	117.69	126.21	-1.30
PC ae C36:1	94.13	138.17	79.35	39.22	81.65	89.92	43.42	72.06	94.59	90.60	74.38	63.57	48.00	59.91	69.54	47.94	53.23	56.98	-1.30
PC ae C36:2	178.93	262.83	145.60	70.97	155.18	173.76	78.79	143.75	212.80	160.17	136.69	118.04	92.07	115.11	125.28	92.28	107.70	118.49	-1.33
PC ae C36:3	71.64	116.22	59.72	29.68	63.99	71.60	31.43	67.68	103.73	68.79	56.00	49.95	37.37	48.70	52.83	36.91	46.57	47.54	-1.38

Supplementary Table S4. Metabolite concentrations (uM) and relative difference in abundance as fold change in the discriminant (from S-plots generated by OPLS-DA) metabolites between G12C and WT KRAS isoforms on monolayer cell-derived xenograft tumors. R# number of biological replicates

Metabolites	WT				G12C				**Fold change G12C vs WT**
	R1	R2	R3	R4	R1	R2	R3	R4	
sugars	95.9	17.4	88.7	101	74.7	49.4	41.6	112	-1.09
Ala	550	314	231	317	523	463	560	600	1.52
Asp	79	46	37	79	49.3	45	69	75.7	-1.01
Gln	203	92.7	115	133	62	106	81.3	96.3	-1.57
Glu	1013	843	597	793	593	540	580	703	-1.34
His	49.7	32.2	28.9	42	39.3	38.7	46.7	39.7	1.08
Ile	41.7	20.1	18.8	28.2	32.9	32.1	34.7	35.3	1.24
Leu	98.7	46.7	49	67.3	75	68	80.7	87.3	1.19
Lys	84.3	62	65.3	58.7	72.7	76.7	107	58.7	1.17
Met	59.3	30.3	28.5	40.3	43.7	34	49.7	53	1.14
Orn	11.5	9.6	9.37	10.4	11.2	21.4	22.4	13.2	1.67
Phe	56	26.8	31.9	37.7	45.7	43	53	51.7	1.27
Pro	224	131	130	149	178	143	171	180	1.06
Ser	63.3	37.3	29.9	38.3	41.7	38	70.3	57.7	1.23
Thr	176	152	127	155	168	204	191	173	1.21
Trp	13.7	8.53	9.67	12.3	14.7	14.8	18.8	14.8	1.43
Tyr	72.3	27.7	35.3	47.7	48	52	52.7	66	1.20
Val	70.7	50.3	40.3	69	75	66.7	93.7	69.7	1.32
Putrescine	3.67	2.73	2.73	2.34	4.73	4.63	3.73	3.27	1.43
Taurine	160	159	160	138	244	209	220	186	1.39
C0	3.19	2.6	3.88	3.39	9.75	6.69	8.12	7.19	2.43
C2	3.54	2.47	3.04	2.83	9.44	7.48	8.01	6.8	2.67
C4	0.388	0.355	0.356	0.304	1.15	0.964	1.49	0.866	3.19

Supplementary Table S5. List of the measurable metabolites using the Biocrates Absolute IDQ p180 kit

METABOLITE CLASS	NUMBER	METABOLITE NAME OR ABBREVIATION	BIOLOGICAL RELEVANCE (SELECTED EXAMPLES)
**AMINO ACIDS**	21	Alanine, arginine, aspartate, citrulline, glutamine, glutamate, glycine, histidine, isoleucine, leucine, lysine, methionine, ornithine, phenylalanine, proline, serine, threonine, tryptophan, tyrosine, valine	Amino acid metabolism, urea cycle, activity of gluconeogenesis and glycolysis, insulin sensitivity, neurotransmitter metabolism, oxidative stress
**CARNITINE**	1	C0	Energy metabolism, fatty acid transport and mitochondrial fatty acid oxidation, ketosis, oxidative stress, mitochondrial membrane damage
**ACYLCARNITINE**	39	C2, C3, C3:1, C3-OH, C4, C4:1, C4-OH, C5, C5:1, C5:1-DC, C5-DC, C5-M-DC, C5-OH, C6, C6:1, C7-DC, C8, C9, C10, C10:1, C10:2, C12, C12-DC, C14, C14:1, C14:1-OH, C14:2, C14:2-OH, C16, C16:1, C16:1-OH, C16:2, C16:2-OH, C16-OH, C18, C18:1, C18:1-OH, C18:2	
**BIOGENIC AMINES**	19	Acetylornithine, asymmetric dimethylarginine, total dimethylarginine, alpha-aminoadipic acid, carnosine, creatinine, histamine, kynurenine, methionine sulfoxide, nitrotyrosine, hydroxyproline, phenylethylamine, putrescine, sarcosine, serotonin, spermidine, spermine, taurine	Neurological disorders, cell proliferation, cell cycle progression, DNA stability, oxidative stress
**LYSO-PHOSPHATIDYLCHOLINES**	14	lysoPC a C14:0/C16:0/C16:1/C17:0/C18:0/C18:1/C18:2/C20:3/C20:4/C26:0/C26:1/C28:0/C28:1	Degradation of phospholipids, membrane damage, signaling cascades, fatty acid profile
**DIACYL-PHOSPHATIDYLCHOLINES**	38	PC aa C24:0/C26:0/C28:1/C30:0/C30:2/C32:0/C32:1/C32:2/C32:3/C34:1/C32:2/C34:3/C32:4/C36:0/C36:1/C36:2/C36:3/C36:4/C36:5/C36:6/C38:0/C38:1/C38:3/C38:4/C38:5/C38:6/C40:1/C40:2/C40:3/C40:4/C40:5/C40:6/C42:0/C42:1/C42:2/C42:4/C42:5/C42:6	Dyslipidemia, membrane composition and damage, fatty acid profile, activity of desaturases
**ACYL-ALKYL-PHOSPHATIDYLCHOLINE**	38	PC ae C30:0/C30:2/C32:1/C32:2/C34:0/C34:1/C34:2/C34:3/C36:0/C36:1/C36:2/C36:3/C36:4/C36:5/C38:0/C38:1/C38:2/C38:3/C38:4/C38:5/C38:6/C40:1/C40:2/C40:3/C40:4/C40:5/C40:6/C42:0/C42:1/C42:2/C42:3/C42:4/C42:5/C44:3/C44:4/C44:5/C44:6	
**SPHINGOMYELINS**	15	SM (OH) C14:1, SM C16:0, SM C16:1, SM C16:1, SM C18:0, SM C18:1, SM C20:2, SM C22:3, SM (OH) C22:1, SM (OH) C22:2, SM C24:0, SM C24:1, SM (OH) C24:1, SM C26:0, SM C26:1	Signaling cascades, membrane damage (eg. neurodegeneration)
**HEXOSE**	1	H1	Carbohydrate metabolism
**TOTAL**	186		

Aa, acyl-acyl; ae, acyl-alkyl; a, lyso; Cx:y, where x is the number of carbons in the fatty acid side chain; y is the number of double bonds in the fatty acid side chain; DC, decarboxyl; M methyl; OH, hydroxyl; PC, phosphatidylcholine; SM, sphingomyeline
